# Distribution of *Clinostomum complanatum* in two commercially important freshwater fish, perch and rudd, in France^☆^

**DOI:** 10.1016/j.fawpar.2025.e00281

**Published:** 2025-08-08

**Authors:** Maureen Duflot, Françoise Pozet, Sophie Le Bouquin, Céline Richomme, Odile Bourgau, Isabel Blasco-Costa, Mélanie Gay

**Affiliations:** a Anses, Laboratory for Food Safety, Boulogne-sur-Mer, France; b JRU Parasite Immunology and Molecular Biology (BIPAR), ENVA-INRAE-ANSES, Animal Health Laboratory, Maisons-Alfort, France; c Jura Departemental Analysis Laboratory, Poligny, France; d Anses, Ploufragan-Plouzané-Niort Laboratory, Epidemiology Health and Welfare Unit, Ploufragan, France; e Anses, Nancy Laboratory for Rabies and Wildlife, Malzéville, France; f Department of Invertebrates, Natural History Museum of Geneva, Geneva, Switzerland

**Keywords:** *Clinostomum complanatum*, Parasite, Prevalence, Zoonoses, Freshwater fish, Emerging trematode, Mitochondrial DNA

## Abstract

Over the last two decades, the popularization of new eating habits and the increase in fish products demand led to a raising risk for consumers due to food-borne parasitic zoonoses. Species of *Clinostomum* Leidy, 1856 are cosmopolitan zoonotic digenetic trematodes. They are present, at the juvenile stage, in numerous freshwater fish. Among them, *Clinostomum complanatum* may induce pharyngitis or laryngitis in humans following consumption of raw fish infected by the metacercariae. In France, the first mention of *Clinostomum* spp. metacercariae on freshwater fish was made in the Durance River in 2008, with almost no data and very obscure conclusions. However, recently, the infection of a batch of wild perch in the Jura in December 2019 was described. The present study provides the first evidence of the extent of the distribution of *C. complanatum* across continental France based on sampling of freshwater fish (European perch and rudd) from different departments. A survey of infection levels was realized on 14 batches of fish collected in eight departments. Fish were sampled by recreational anglers or pond fish farmers. They were dissected for the presence of *C. complanatum* metacercariae. All metacercariae were identified based on molecular analyses on mtDNA *cox1* gene fragment and rDNA *28S* gene fragment. Encysted metacercariae were found in five departments and in 113 out of 526 sampled fish. Intensity of infection ranged from 4 to 25 parasites per fish. All metacercariae were identified as *C. complanatum.* Phylogenetic and haplotype network analyses reported low genetic diversity in French *C. complanatum* individuals on mtDNA *cox1* marker indicating the absence of distinct populations in the French territory. French specimens shared common mtDNA haplotypes with *C. complanatum* specimens from Italy. This study confirmed the presence of this zoonotic trematode species in freshwater consumed fish, and expanded the known geographical distribution area in France. Moreover, the high intensity and prevalence recorded suggested it could represent a hazard both to human and animal health. We discuss how future research should address the zoonotic risk of this parasite and ensure the health safety of fish-based products and new consumer habits.

## Highlights

• Confirmation of *C. complanatum* presence in consumed fish from 5 French departments
• Prevalence of infected fish batches varied between 3.8 and 90.6 %
• Mean intensity per infected fish batch varied between 1.8 and 5.3 parasites per fish
• Identification of metacercariae based on sequencing of *cox1* and rDNA *28S* fragments
• Shared common haplotypes between French and Italian samples

## Graphical abstract

**Figure:**
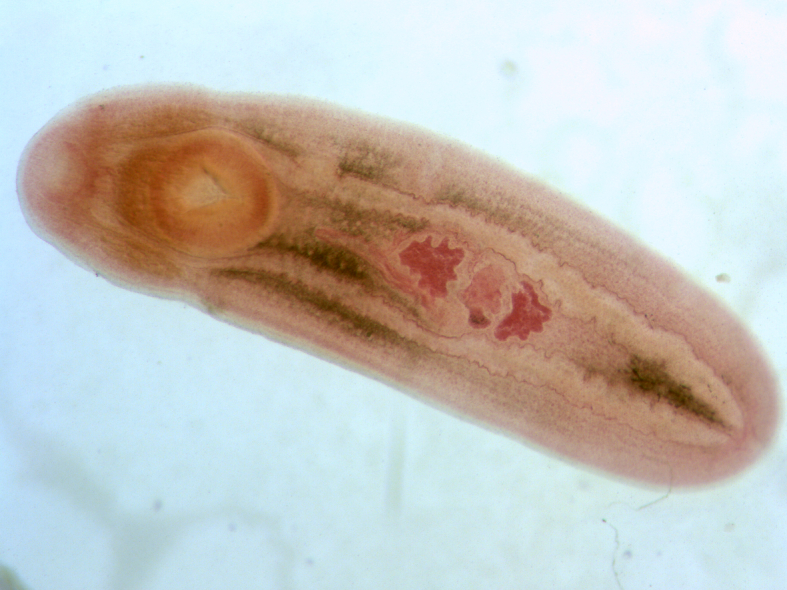

## 1 Introduction

Considering parasites of public health importance in fishery products, EFSA (2010) recommended to acquire data on their life cycle, geographical and seasonal distribution, prevalence, intensity, and anatomical location. Moreover, nowadays, geographical limits and populations at risk are currently expanding and changing related to factors such as growing international markets, evolution of consumption behavior, demographic changes or global warming (Dupouy-Camet et al., 2020; EFSA, 2010; EFSA BIOHAZ PANEL et al., 2024). This has recently led to a growing interest in trematode infections highlighted by the need to clarify the current epidemiology of these helminth infections (Chai and Jung, 2024).

*Clinostomum* Leidy, 1856 is a genus of digenetic trematodes widely distributed in the world, with more than 20 species reported from Europe (Ermakova et al., 2024; Esposito et al., 2024), Asia (Ika and Anshary, 2024; Suwancharoen et al., 2023), Africa (El-Khayat et al., 2024; Obayemi et al., 2023), Oceania (Shamsi et al., 2023), North (Nguyen et al., 2024) and South America (Di Cesare et al., 2024; Velázquez-Urrieta et al., 2024). They are endoparasites with complex life cycles involving three hosts. Commonly they use freshwater snails from the family Lymnaeidae as first intermediate host (Gustinelli et al., 2010). The small forked-tail cercariae are released in the aquatic environment, free-living but with a short lifespan before they infect the second intermediate host, typically a fish or amphibian, in which the cercariae develop into the juvenile metacercariae stage. Metacercariae are large and often yellow due to the colour of their gut contents, they are found in the tissues (e.g. dermis, muscles, head, gill arch) or body cavity of freshwater fish and are often called ‘yellow grubs’ (Gustinelli et al., 2010; Matthews and Cribb, 1998). Many fish species have been reported as second intermediate hosts (Bullard and Overstreet, 2008; Rochat et al., 2025; Shafiq et al., 2023). Piscivorous birds from the families Ardeidae, Phalacrocoracidae and Anhingidae as well as mammals have been reported as definitive hosts (Briosio-Aguilar et al., 2018; Bullard and Overstreet, 2008). Adults inhabit the oral cavity, pharynx or esophagus of definitive hosts, which ingest second intermediate hosts (Kanev et al., 2002; Matthews and Cribb, 1998). Additionally, *C. complanatum* is considered as a zoonotic parasite for public health (Matthews and Cribb, 1998; Shafiq et al., 2023). Human infections with adult clinostomids have been reported in Korea (Kim et al., 2023; Chung et al., 1995), in Japan (Hara et al., 2014; Yamashita, 1938) and Russia (Ermakova et al., 2024). Humans can be infected after eating raw or undercooked fish, leading to Halzoun syndrome (Hara et al., 2014; Kim et al., 2023). Infection sites in humans involve the pharynx, arytenoid region, posterior oropharyngeal wall, and lateral lymphatic band, causing discomfort in the throat, pain in swallowing, bloody phlegm, and fever (Hara et al., 2014, Kim et al., 2023).

Comparison of mitochondrial and ribosomal DNA and morphological differentiations demonstrated the need to reorganize *Clinostomum* taxonomy. Caffara et al. (2020) reported 16 valid species. The most common species were *C. complanatum* encountered in Europe and Africa, *C. sinensis* reported in Asia (Locke et al., 2019) and *C. marginatum* that ranges throughout North America (Dzikowski et al., 2004) to Mexico (Scholz et al., 1995).

Few data are available regarding the distribution of *Clinostomum* spp. in European freshwaters ecosystems. *Clinostomum complanatum* have been recorded in some mollusks of the Lymnaeidae in Italy (Kalantan et al., 1987), in a range of fish species in Czech Republic (Kadlec et al., 2003), Hungary (Antal et al., 2015), Italy (Locke et al., 2019; Locke et al., 2015), Poland (Grababda Kazubska, 1974), Romania (Cojocaru, 2009), and Slovakia (Fedorcak et al., 2019), and in some amphibians and birds from Germany (Yamaguti, 1958), Italy (Caffara et al., 2014), Poland (Grababda Kazubska, 1974) and Slovakia (Yamaguti, 1971). The former observations of *C. complanatum* on European perch in France were made in 2008 in the Durance River (Davidova et al., 2008), in 2019 (Rochat et al., 2025) in the Doubs River and in 2023 in Paula Reservoir in Corsica (Esposito et al., 2024).

The changing habits of fish consumption introduce new risks to human health (Brauge et al., 2022; Dupouy-Camet et al., 2020; Fried et al., 2004). In this study, following former data (Esposito et al., 2024; Rochat et al., 2025) the European perch was selected as indicator of the distribution of *C. complanatum* and zoonotic risk based The primary aim was to characterize the distribution of this parasite in France. Moreover, sequences from newly obtained samples and preexisting data allowed identification of species and assessment of genetic diversity.

## 2 Material and methods

### 2.1 Collection of fish and parasitological investigations

European perch *Perca fluviatilis,* Linnaeus, 1758 was targeted in this study. All fish were collected from April 2021 to September 2023 in France. The initial protocol included the collection of 30 to 50 individuals for each sampling batch. One batch always originated from a single sampling site (section of a river, pond, etc.) but several batches could come from the same department (France is geographically divided into 94 administrative departments, each administered by an elected departmental council; most of the samples were obtained through departmental recreational fishing associations; thus, the department is the geographical division considered throughout the present work). The fish batches were obtained from recreational fishermen or professional fish farm contributing voluntarily to this research project. Abiding to the confidentiality agreement with the contributors of fish samples, the precise location of the batches analyzed will not be provided and the data was displayed and analyzed at the departmental level. All fish were frozen at −20 °C rapidly after capture, kept frozen until analysis at lab. Fish were unfrozen slowly overnight at 1 °C.

Standard total length, weight and sex were recorded for each fish before parasitological examination. Fish were dissected by peeling the skin and filleting the flesh. Fillets and skin were first observed by the naked eye and then on candling table. All muscles were examined for encysted parasites. The presence and localization of cysts on both sides of fish was annotated. Metacercariae were collected and fixed in absolute ethanol (99 %) for molecular analyses.

Three classical parasitological descriptors, prevalence, intensity and abundance, were used in the present study following Bush et al. (1997) to describe the distribution of *C. complanatum* in the fillets. The data were analyzed with Microsoft Excel (Microsoft Corporation, 2018) and visualized with QGIS, 2025 3.26.3-Buenos Aires (QGIS.org). All data produced by this study, collected formerly by project partners and unpublished or published (Davidova et al., 2008; Rochat et al., 2025; Esposito et al., 2024) were synthesized to produce a global view of the knowledge on the distribution of *C. complanatum* in France.

### 2.2 Identification of metacercariae

Molecular identification was carried out following the protocol of DNA extraction, PCR amplification targeting fragments of mtDNA *cox1* gene and of the large ribosomal subunit *28S* rRNA gene, Sanger sequencing and bioinformatics analyses described by Rochat et al., 2025.

For each sequence, a basic local alignment search tool (BLAST) (Altschul et al., 1997) was carried out with the use of the NCBI database after visualization in BioEdit 7.0.9.0 software and assembling in Geneious 2023.2.1 software (https://www.geneious.com).

### 2.3 Phylogenetic analysis and genetic diversity of *C. complanatum* populations

Five genomic DNA per fish sampling batch were used for phylogenetic and haplotype network constructions. Available sequences belonging to Clinostomidae family were retrieved from GenBank and aligned using MAFFT alignment of Geneious 2023.2.1 software. Phylogenetic trees were constructed for both the mtDNA *cox1* and *28S* genes using CIPRES Science Gateway (Miller et al., 2010). Maximum Likelihood (ML), and Bayesian inference (BI) methods were used for separate and combined nucleotide data sets with outgroups sequences. ML analysis were conducted using RAxML-HPC2 8.2.12 (Stamatakis et al., 2008) and using 1000 bootstrap replications. BI phylogenies were carried out using MrBayes 3.2.6 (Rohde and Littlewood, 2005). Two independent runs were performed for 10,000,000 generations and sampled every 500th generation. The burn-in was set for the first 25 % of the sampled trees. The final trees were drawn using FigTree software version 1.4.0 (http://tree.bio.ed.ac.uk/software/figtree/). Estimations of the genetic diversity of *C. Complanatum* were inferred from mtDNA *cox1* gene data sets comprising the newly generated sequences, those available from other specimens in France and in the literature from other parts in Europe and the following parameters were calculated: number of haplotypes (Nh), nucleotide diversity (p), haplotype diversity (Hd), average number of differences (K), number of polymorphic sites (S), using DnaSP 6.12.03 software (Rozas et al., 2017). The haplotype network for the mtDNA *cox1* gene was built using PopART 1.7 software (Leigh and Bryant, 2015).

## 3 Results

### 3.1 Sampling

14 batches and a total of 526 fish were collected in eight different departments of France (Table 1), with a mean of 37 fish per batch (min. 10 – max. 39). Despite this study mainly focused on the European perch, one batch of rudd, *Scardinius erythrophthalmus* (Linnaeus, 1758) was collected and two pumpkinseed individuals, *Lepomis gibbosus* (Linnaeus, 1758) were found in the batch originating from the Nord department (department number 59). Mean total length of perch and rudd was 15.4 ± 0.4 (8.5–38.0) cm and 10.3 ± 0.7 (7.0–21.0) cm respectively. Mean total weight of perch and rudd was 61.6 ± 6.5 (5.9–752.8) g and 16.1 ± 5.6 (3.5–123.7) g respectively.

**Table 1:** Description of the batches of fish: geographical origin (by department, an administrative division of the French territory), fish species, number of individuals and biometric data of fish specimens, including mean weight and length of fish sampled per department. SD: standard deviation. Min: minimum. Max: maximum.

Fishing department (administrative number and name)	Number of batches	Species	Number of fish	Mean weight of fish in g ± SD (min-max)	Mean total length of fish in cm ± SD (min-max)
21 - Côte d'Or	1	European perch	35	28.3 ± 2.6 (15.5–47.1)	13.6 ± 0.4 (11.6–16.3)
25 - Doubs	1	European perch	10	179.3 ± 134.7 (13.3–752.8)	20.2 ± 6.9 (10.6–38.0)
36 - Indre	3	European perch and Rudd	64 53	133.9 ± 24.2 (33.0–474.6) 16.1 ± 26.6 (3.5–123.7)	20.1 ± 2.4 (12.5–31.2) 12.0 ± 2.7 (7.0–99.6)
39 - Jura	2	European perch	72	61.2 ± 12.8 (5.9–254.4)	15.2 ± 0.7 (8.6–24.0)
57 - Moselle	1	European perch	50	17.0 ± 1.3 (10.1–31.4)	11.5 ± 0.2 (10.0–14.0)
59 - Nord	1	Perch (European and pumpkinseed)	18	67.4 ± 30.4 (12.0–286.1)	15.7 ± 2.1 (8.5–27.6)
71 - Saône et Loire	3	European perch	106	45.1 ± 7.1 (9.0–189.7)	14.6 ± 0.7 (8.6–24.2)
74 - Haute Savoie	2	European perch	118	55.5 ± 7.8 (10.5–278.6)	15.8 ± 0.6 (10.1–25.6)
TOTAL	14	–	526	57.0 ± 6.0 (3.5–752.8)	15.1 ± 0.5 (7.0–99.6)

### 3.2 Infection levels of freshwaters fish and distribution of *C. complanatum*

Of the 526 fish examined, 113 fish were infected. European perch and rudd were found infected with a prevalence of 13.7 and 90.6 % respectively. The two pumpkinseed individuals analyzed were uninfected. The cysts of parasites were mainly observed on the caudal region, the dorsal area above the lateral line, on the gill operculum and behind the pectoral fins in both European perch and rudd. No difference of infection was noticed between right and left side of the fish or between female and male.

**Fig. 1:**
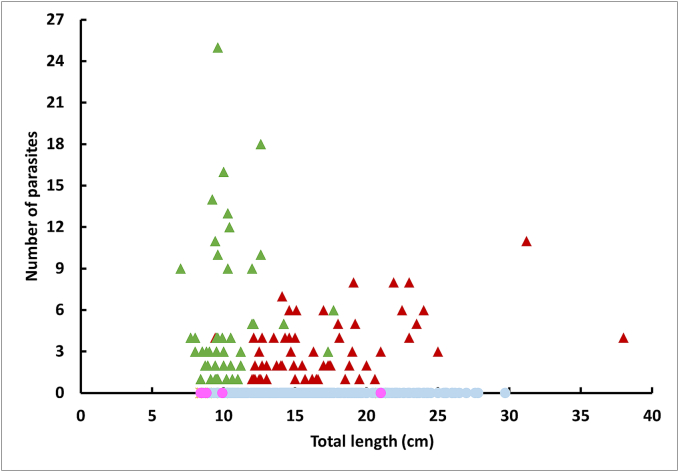
Clinostomum complanatum infection per fish, in perch (infected:  and uninfected: ) and in rudd (infected:  and uninfected: ) as a function of fish total length.

By looking at the weights and sizes of the infected fish as a whole (Table 2 and Fig. 1), the entire range of sizes and weights of the sample was parasitized without any pattern between these biometric data and infection levels.

**Table 2:** Biometry of fish infected with *C. complanatum* metacercariae and parasitological descriptors (prevalence, intensity and abundance) per French department (an administrative division of the French territory) (CI: Confidence interval).

Fishing department	Species	Mean weight of infected fish in g ± CI (min-max)	Mean total length of infected fish in cm ± CI (min-max)	Prevalence (%)	Mean intensity ± CI	Mean abundance ± CI	Maximum number of cysts per fish
21 - Côte d'Or	Perch	29.0 ± 3.3 (17.4–47.1)	13.7 ± 0.4 (12.0–16.3)	71.4	2.7 ± 0.6	1.9 ± 0.5	7
25 - Doubs	Perch	217.5 ± 157.9 (44.3–752.8)	25.3 ± 7.4 (15.0–38.0)	80.0	3.8 ± 1.7	3.0 ± 1.5	8
36 - Indre	Perch Rudd	100.4 ± 45.3 (36.7–425.8) 14.5 ± 4.2 (3.5–79.5)	18.6 ± 0.9 (14.6–31.2) 10.2 ± 0.6 (7.0–17.7)	25 90.6	4.1 ± 1.5 5.3 ± 1.5	1.0 ± 0.8 4.8 ± 1.3	11 25
39 - Jura	Perch	95.9 ± 25.9 (21.5–191.0)	18.3 ± 0.8 (12.7–22.5)	16.7	2.3 ± 1.0	0.4 ± 0.4	6
57 - Moselle	Perch	–	–	0.0	0.0	0.0	0
59 - Nord	Perch	–	–	0.0	0.0	0.0	0
71 - Saône-et-Loire	Perch	24.5 ± 22.7 (9.0–57.7)	11.9 ± 0.6 (9.4–16.2)	3.8	1.8 ± 1.5	0.1 ± 0.3	4
74 - Haute-Savoie	Perch	–	–	0.0	0.0	0.0	0
TOTAL	–	53.3 ± 16.6 (3.5–752.8)	14.2 ± 0.9 (7.0–99.6)	21.5	1.0 ± 0.5	0.9 ± 0.2	25

**Fig. 2:**
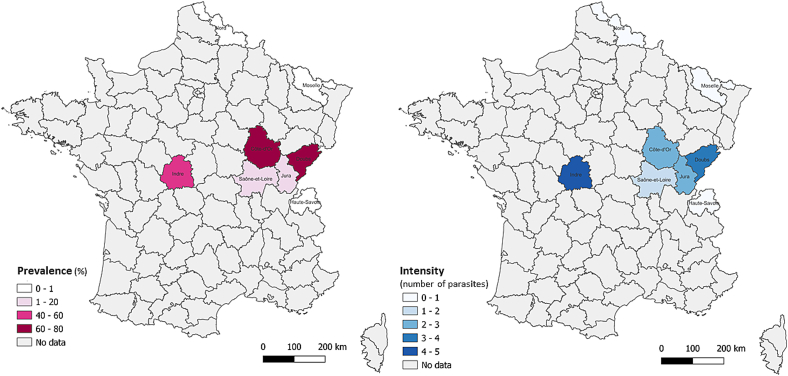
Prevalence (left) and intensity (right) of *Clinostomum complanatum* infection in fish sampled in eight departments in France.

Infection by encysted metacercariae was observed in five out of the eight departments sampled (Table 2). The prevalence (from 3.8 % to 90.6 % of infected fish) was highly variable between the five departments where the parasite was found (Table 2). Considering all batches, the highest prevalence was observed for the only rudd batch, coming from Indre, with 90.6 % of infected fish. Considering perch sampling, the highest prevalence was detected in the Doubs and Côte d'Or departments with more than 70 % of infected fish (Fig. 2), followed by Indre (25 %), Jura (16.7 %) and Saône-et-Loire (3.8 %). The batches coming from the Nord, Moselle and Haute-Savoie departments were not found infected.

Infection intensity and abundance followed the same trends as the proportion of infected fish (Table 2 and Fig. 2). The mean intensities of the different fish species also varied by department. For the rudd, the mean intensity was 4.1 parasites, whereas for the European perch, the intensities ranged from 1.8 to 5.3 parasites. The highest values of intensity and abundance were observed for the rudd batch, with 5.3 ± 1.5 and 4.8 ± 1.3 respectively. Perch from the Doubs and Indre had the highest mean intensities (3.8 ± 1.7 and 4.1 ± 1.5 respectively) and perch from the Doubs and Côte d'Or had the highest abundances (3.0 ± 1.5 and 1.9 ± 0.5 respectively). Côte d'Or and Jura batches had intermediate intensities (2.7 ± 0.6 and 2.3 ± 1.0 respectively), and Côte d'Or and Indre had intermediate abundances (1.9 ± 0.5 and 1.0 ± 0.8 respectively). The lowest intensity and abundance were observed in Saône-et-Loire (1.8 ± 1.5 and 0.1 ± 0.3 respectively).

Heterogeneity of infection within the same department was observed, as shown for example by the maximum number of cysts per perch in the Indre (11) compared with the parasite intensity (4.1 parasites) and abundance (1.1 parasites). The same observation can be made for fish sampled in the Jura. Again, the rudd batch had the highest value for the maximum number of metacercariae per fish with 25.

### 3.3 Parasite identification and genetic diversity estimates

In total, *28S* sequences were obtained from 234 specimens and mtDNA *cox1* sequences were obtained from 292 specimens. Sequences of both markers were obtained from 229*C. complanatum* metacercariae. The different sequences obtained from the different departments were deposited on GenBank (PQ656398- PQ656417 for mtDNA *cox1* and PQ658174-PQ658186 for *28S*). The consensus sequences of the mtDNA *cox1* gene analyzed by BLAST search gave an identity between 96 and 100 % with *C. complanatum* (MK811210- MT603881). The consensus sequences of the *28S* rDNA gene evaluated against BLAST database gave a range from 95.6 to 100 % identity with *C. complanatum* (OP681143 or MK814187).

**Fig. 3:**
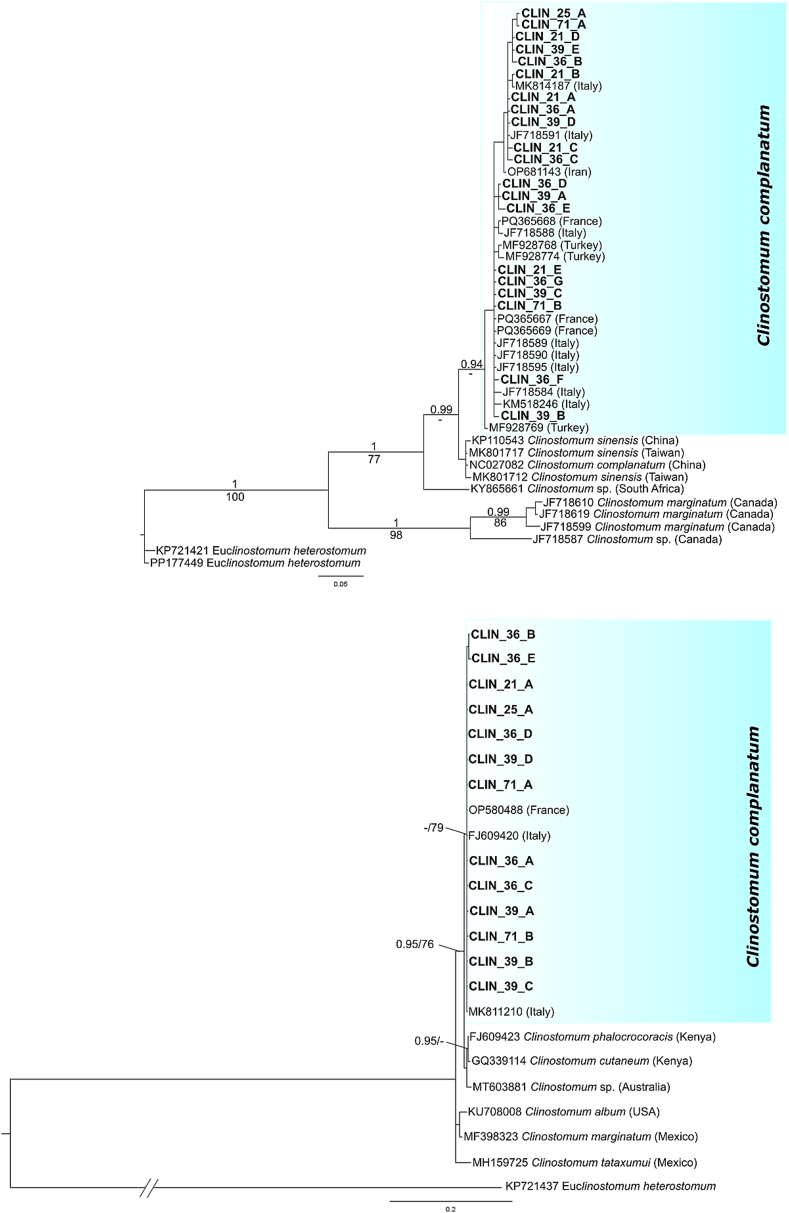
Maximum likelihood trees based on the *cox1* mtDNA (above) and *28S* (bottom) datasets of *Clinostomum* species, including newly generated sequences of *Clinostomum complanatum* and available sequences from GenBank (shaded in blue). Nodal support is provided as posterior probabilities (number above the branches or only number; not shown if <0.9) and maximum parsimony bootstrap percentages (number below; not shown if <70 %). (For interpretation of the references to colour in this figure legend, the reader is referred to the web version of this article.)

The mtDNA *cox1* and *28S* datasets used in the phylogenetic analyses had a total length of 480 bp and 1169 bp, respectively. Bayesian Inference and Maximum Likelihood phylogenetic analyses produced congruent trees despite most nodes showing relative low support values, with some exceptions (Fig. 3). The phylogenetic trees based on the *28S* rRNA gene region displayed only relative support for the clade comprising three *C. complanatum* sequences together with our newly obtained sequences from France (BS 79 %), and the clade comprising sequences representative of *C. complanatum* and *Clinostomum phalacrocoracis*, *C. cutaneum* and *Clinostomum* sp. from Australia (BS 76 %, PP 0.95). The BI tree also showed support for the clade comprising *C. phalacrocoracis*, *C. cutaneum* and *Clinostomum* sp. from Australia which appeared as sister to *C. complanatum* although unsupported. The phylogenetic trees based on the mtDNA *cox1* marker depicted all new sequences from France clustering together with sequences of *C. complanatum* available from Genbank (BS 58 %, PP 1) in a monophyletic clade. More specifically, newly generated specimens grouped with *C. complanatum* specimens of metacercariae from France, Italy, Iran, Turkey and Taiwan (see Table 3 for detailed origins). *C. sinensis* appeared as sister species to *C. complanatum* (BS 54 %, PP 1). Other species of *Clinostomum* spp. occupied basal positions in trees.

**Table 3:** *28S* and *mtDNA cox1* sequences from the literature and used as references in the phylogenetic analyses (Fig. 3): target gene, parasite and host species, geographical origin, references from GenBank and the literature.

Gene	Species	Host species	Origin	GenBank accession	Reference
*28S*	*Clinostomum album*	*Ardea alba*	USA	KU708008	Rosser et al. [68]
	*Clinostomum complanatum*	*Perca fluviatilis*	France	PQ658174-PQ658186	This study
			France	OP580488	Rochat et al. [19]
		*Squalius cephalus*	Italy	MK811210	Locke et al. [26]
		*Wild barbel*	Italy	FJ609420	Gustinelli et al. [15]
	*Clinostomum cutaneum*	*Ardea cinerea*	Kenya	GQ339114	Gustinelli et al. [15]
	*Clinostomum marginatum*	*Ardea alba*	Mexico	MF398323	Hernández-Mena et al. [69]
	*Clinostomum phalacrocoracis*	*Ardea cinerea*	Kenya	FJ609423	Gustinelli et al. [15]
	*Clinostomum* sp.	*Rana catesbeiana*	USA	AY222176	Olson et al. [70]
	*Clinostomum tataxumui*	*Hypseleotris* sp.	Australia	MT603881	Rochat et al. [71]
		*Bagre marinus*	Mexico	MH159725	Briosio-Aguilar et al. [72]
	Outgroup taxa *Euclinostomum heterostomum*	Cichlids	Israel	KP721437	Caffara et al. [73]
mtDNA *cox1*	*Clinostomum complanatum*	*Barbus barbus*	Italy	JF718591; JF718595	Caffara et al. [25]
		*Carassius auratus*	China	NC027082	Chen [74]
		*Lepomis gibbosus*	Italy	JF718589	Caffara et al. [25]
		Newts	Italy	KM518246	Locke et al. [26]
		*Nycticorax nycticorax*	Iran	OP681143	Monnens et al. [66]
		*Perca fluviatilis*	France	PQ656398- PQ656417	This study
			France	PQ365667- PQ365669	Rochat et al. [19]
		*Squalius cephalus*	Turkey	MF928774; MF928768; MF928769	Simsek et al. [67]
		*Squalius cephalus*	Italy	MK814187	Locke et al. [26]
		*Squalius cephalus*	Italy	JF718588; JF718590	Caffara et al. [25]
	*Clinostomum marginatum*	*Ambloplites rupestris*	Canada	JF718599	Caffara et al. [25]
		*Lepomis gibbosus*	Canada	JF718619	Caffara et al. [25]
		*Perca flavescens*	Canada	JF718610	Caffara et al. [25]
	*Clinostomum sinensis*	unknown	China	KP110543	Locke et al. [40]
		unknown	Taiwan	MK801712	Locke et al. [40]
		*Candidia barbata*	Taiwan	MK801717	Locke et al. [26]
	*Clinostomum* sp.	*Barbus barbus*	Italy	JF718584	Caffara et al. [25]
		*Barbus trimaculatus*	South Africa	KY865661	Caffara et al. [25]
		*Rana pipiens*	Canada	JF718587	Caffara et al. [25]
	Outgroup taxa *Euclinostomum heterostomum*	Cichlids *Nile tilapia*	Israel Unknown	KP721421 PP177449	Caffara et al. [73] Mahdy O. (unpublished)

Genetic diversity of *C. complanatum* specimens was estimated based on partial sequences for the mtDNA *cox1* gene. Intraspecific sequence variability ranged from 0.21 to 1.46 % on that marker. Based on the sequences newly obtained for *C. complanatum* metacercariae specimens from different fish species from continental France, parasites exhibited 12 distinct haplotypes (*Nh*). Among these sequences, 14 polymorphic sites (S) were recorded. Sequences showed very low haplotype diversity (*Hd* = 0.882) and low nucleotide diversity (*pi* = 0.00682). The average number of nucleotide differences was also low (*K* = 3.286). By adding the available sequences of other French specimens of *C. complanatum* to those from the present study, the number of haplotypes increased to 13, with 15 polymorphic sites (S). Haplotype diversity, nucleotide diversity and average number of nucleotide differences remained very low (*Hd* = 0.873, *pi* = 0.00674 and *K* = 3.236). Further addition of *C. complanatum* specimens from other origins increased the number of haplotype to 41 but nucleotide diversity remained low with only 46 polymorphic sites. The haplotype diversity (*Hd* = 0.893), nucleotide diversity (*pi* = 0.02309)) and average number of nucleotide differences (*K* = 8.468) only slightly increased.

**Fig. 4:**
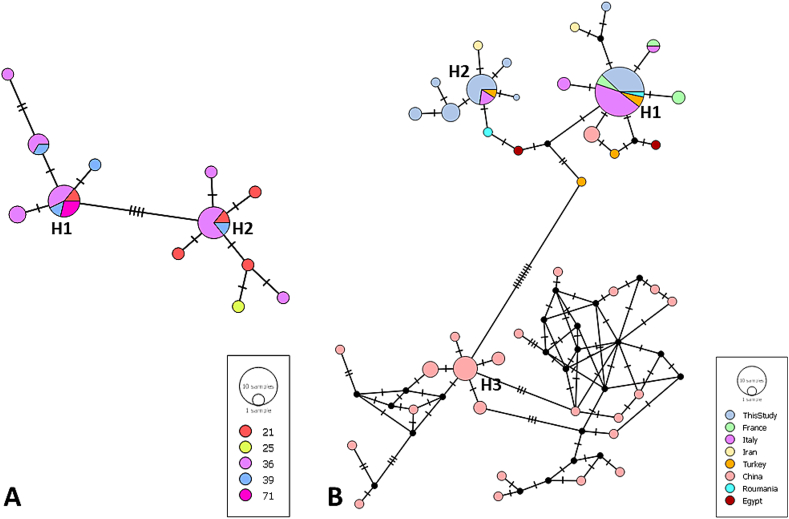
Haplotype networks of *Clinostomum complanatum* based on the *cox1* gene sequences from the different departments of France collected in this study (A; *n* = 29 sequences, 480 pb) and based on *cox1* gene sequences of this study and available data in literature using different countries as grouping variable (B; *n* = 97 sequences, 378 pb). Included departments: 21 - Côte d'Or; 25 – Doubs; 36 – Indre; 39 – Jura; 71 - Saône-et-Loire.

Two median joining network reconstructions based on the mtDNA *cox1* showed the relationships among the 29 sequences of *C. complanatum* from France (Fig. 4 A), and among our sequences and other 97 available sequences from the Palearctic (Fig. 4 B, Table 3). Among the French sequences, two main haplotypes were detected (Fig. 4 A), H1 was shared by specimens of 4 out of the 5 departments and H2 was shared by specimens of 3 out of the 5 departments. The specimen from the Doubs (25) had a unique haplotype, not found in any other locality*.* The haplotype network for *C. complanatum* mtDNA *cox1* sequences from the Palearctic (Fig. 4 B), showed three major haplotypes, H1 was shared by specimens of *C. complanatum* from our study, others in France, from Romania, from Turkey and from Italy, whereas H2 was shared by specimens from our study and those from Italy and Turkey. No haplotypes were shared between the specimens from the western Palearctic and those from China.

### 3.4 Geographical observations of *C. complanatum* from 2019 to 2024 in France

**Fig. 5:**
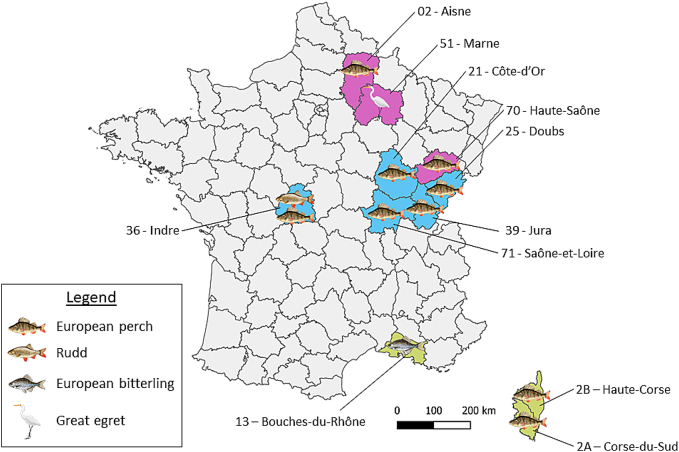
Distribution of *Clinostomum complanatum* in France from 2019 to 2024 including data from the present study (blue), as well as data provided by project partners (pink) and recent literature (green) (Esposito et al., 2024). Illustrations from OPSN (2024) and biorender.com under licence KG272LNSVU. (For interpretation of the references to colour in this figure legend, the reader is referred to the web version of this article.)

The synthesis of various reports of *C. complanatum* obtained for this study and data provided by project partners allowed to create a preliminary map of the distribution of these parasites across France (Fig. 5). Most of the current observations are focused on the second intermediate host. Additionally, the dissection of an egret *Ardea alba* parasitized by *C. complanatum* from the Marne department was also documented (F. Pozet, pers. comm.). Thus far, two fish species, the European perch (*Perca fluviatilis*) and rudd (*Scardinius erythrophthalmus*) were found infected in France.

## 4 Discussion

This study provides a preliminary attempt to elucidate the distribution of *C. complanatum* in French freshwater ecosystems, with a focus on European perch, one of the most appreciated freshwater fish species. Metacercariae of *C. complanatum* were found in fish from 5 out of 8 sampled departments in continental France, suggesting a wider distribution of this parasite in France than previously described, meaning the Durance river, the Corsica and Jura departments (Esposito et al., 2024; Davidova et al., 2008; Rochat et al., 2025). The prevalence seemed to significantly vary in the Jura department (16.7 %, over 2 sites) over a span of two years since it was much lower than the 97 % previously reported on European perch sampled at Orchamps, in a canal adjacent to the Doubs river in the Jura (Rochat et al., 2025). The presence of this parasite had also been formerly observed in two other geographical areas, namely the Aisne and Haute-Saône departments (Pozet & Blasco-Costa, unpublished data, Fig. 5). In the present study, *C. complanatum* has been observed in four additional departments, namely Indre, Saône-et-Loire, Doubs and Côte-d'Or in both rivers and ponds. To sum up, *C. complanatum* was mainly observed in the East of France, with more partial data from the North (Department Aisne and Marne), the Center (Department Indre) and Corsica. Intensities were quite variable, ranging from one to 11 for the perch and from one to 25 for the rudd. The highest number of metacercariae counted was 25 cysts for one fish, with cysts not randomly distributed on the fish. As noted earlier by Rochat et al. (2025) and Kalantan et al. (1987), the opercula and muscles near dorsal fins of the fish seemed to be the preferred regions considering only fillets and skin for both the rudd and the perch. The two pumpkinseed individuals obtained in our sampling were uninfected. Both rudd and perch had already been reported as infected in Turkish and Romanian freshwaters (Simsek et al., 2018; Cojocaru, 2009; Locke et al., 2019).

Phylogenetic analyses corroborated the molecular identification by BLAST of the present specimens as *C. complanatum*. Phylogenetic analysis and haplotype network constructions displayed high levels of relation between the present samples and Rochat's specimens on mtDNA *cox1* gene (Rochat et al., 2025) with low genetic diversity between the present and former mtDNA *cox1* sequences. These results are in concordance with former analyses (Juhásová et al., 2025). The haplotype network reconstructions confirmed the similarity between the sequences of *C. complanatum* from France (this study and Rochat et al. (2025)) and Italy, Iran and Turkey. The sequences of this study thus contribute to the mapping of the population of *C. complanatum* in the western Palearctic. Regarding the number of analyzed sequences and the geographical span, the diversity was still very low, hinting the absence of different populations. Furthermore, no homology was observed with haplotypes from China. This is in accordance with the distinction of *C. sinensis* in East Asia and *C. complanatum* in Europe discussed by Locke et al. (2019), Juhásová et al. (2025). As previously highlighted by Locke et al. (2019), morphological description combined with molecular analyses, could be useful to compare results.

Former observations and the results of the present study led to recommend further, broader investigations, to better characterize the presence of *Clinostomum* spp. in France. Environmental factors such as water temperature or flow, as well as habitat characteristics, fish communities and host should be taken into account in future research, since digenetic trematode like *Clinostomum* could be affected by these abiotic parameters as well as by global change (changes in temperature, but also precipitation, eutrophization etc.). It would be appropriate for further research to consider also the seasonality in *C. complanatum* infection. Indeed, former prevalence and intensity studies observed seasonal variations for *Clinostomum* (El-Kabany et al., 2023; Kalantan et al., 1987). Considering biotic factors, accumulation of parasites with age of fish could be hypothesized too; however, so far, no data are available on the *C. complanatum* metacercariae lifespan. From an ecological point of view, some exotic fish species have been introduced in native ecosystems for recreational fishing or clandestinely as discussed by Esposito et al. (2024). Thus, these practices of re-stocking with or without previous dry period need to be recorded with information on the species, age, quantity and origin of the fish. This will be helpful in the understanding of the dissemination of these parasites at national, European and international scale.

This first map of *C. complanatum* in freshwaters in France would also need to be completed by investigations on the other stages and hosts of its life cycle to understand the circulation of these parasites in their ecosystem. Even though some mollusks were described as host of *C. complanatum*: *Radix auricularia swinhoei* in Taiwan, *Radix auricularia coreana* in Korea and, *Helisoma antrosum* and *Helisoma campanulatum* in the USA (Dias et al., 2003), no data are available for France. Ideally, the infection in the bird definitive host should also be taken into account, for monitoring and understanding all the life cycle of *C. complanatum*, since the large dispersal ability of this host can have a major impact on the emergence of new foci of *C. complanatum* in France and elsewhere in Europe. However, studies of wild and mostly protected species are quite difficult or even impossible to set up.

Our public call to obtain samples from the recreational and professional fishers in the sector resulted in a limited geographic extent of the sampling achieved. Thus, the knowledge on the distribution of *C. complanatum* at the national scale is still incomplete. Since this parasite, poorly known in France, had only barely been previously described in this area, its presence may cause concern to the professional, recreational fishermen, and fish farmers because it could impose new constraints such as the necessity to declare to hygiene services the presence of this parasite in their products. These concerns may explain their smaller contribution than expected.

The concern regarding the zoonotic potential of *C. complanatum*, coupled with the importance of fisheries, require a thorough investigation. With the evolution of consumption habits, especially regarding the consumption of raw or undercooked fish products, some freshwater fish species become more and more popular such as *P. fluviatilis* (Jankowska et al., 2007; Pimakhin et al., 2015). The absence of cases in Europe might be due either to the time lap between the changing dietary habits and the observation of human cases, as hypothesized by Scaramozzino et al. (2018) for *Opisthorchis felineus* in Italy. Consumers and professionals should be aware and informed to consume raw *P. fluviatilis* in a safe manner following the EU regulation and Anses recommendation to consumers (ANSES, 2013; European Parliament, 2004). So far, no research have confirmed the efficacy of freezing or heating on inactivation of *C. complanatum*. Unlike parasites from marine fish like such as the nematode *Anisakis*, for which cooking and freezing guidelines exist (EFSA BIOHAZ PANEL et al., 2024; European Parliament, 2004; Gay and Verrez-Bagnis, 2024), clinostomid trematodes guidelines and resistance data are lagging behind. Further research should be carried out to address this need and ensure the sanitary safety of these new fish-based products.

To conclude, this study provides a more extended view of the distribution and genetic diversity of *C. complanatum* in French freshwater ecosystems, highlighting a potential zoonotic risk. However, in addition to data on safety measures (freezing, cooking conditions) and information campaigns on *Clinostomum* to raise awareness among freshwater fishery professionals, further studies should be carried out in France and in the observed infected regions to investigate a wider variety of host species and to complete the missing geographical data in order to better assess the potential risk for the consumer and the aquaculture sector.

## Availability of data and materials

All raw data on infection levels as well as DNA from metacercariae may be obtained upon request to the corresponding author.

## CRediT authorship contribution statement

**Maureen Duflot:** Writing – original draft, Methodology, Investigation, Formal analysis, Data curation. **Françoise Pozet:** Writing – review & editing, Formal analysis, Conceptualization. **Sophie Le Bouquin:** Writing – review & editing, Conceptualization. **Céline Richomme:** Writing – review & editing, Conceptualization. **Odile Bourgau:** Methodology, Investigation, Data curation. **Isabel Blasco-Costa:** Writing – review & editing, Formal analysis, Data curation, Conceptualization. **Mélanie Gay:** Writing – review & editing, Methodology, Investigation, Funding acquisition, Data curation, Conceptualization.

## Ethics approval and consent to participate

Not applicable.

## Fundings

This research was supported by a grant from French Agency for Food, Environmental and Occupational Helth & Safety (Anses) in the frame of the project ‘Exploratory epidemiological study of the distribution of *C. complanatum*, a zoonotic trematode parasite, in perch, a commercially important freshwater fish’ (ClinExplor).

## Declaration of competing interest

The authors declare the following financial interests/personal relationships which may be considered as potential competing interests:

Melanie Gay reports financial support was provided by 10.13039/501100007546Anses. If there are other authors, they declare that they have no known competing financial interests or personal relationships that could have appeared to influence the work reported in this paper.
